# Supplementary figures and images for: Facial asymmetry and midsagittal plane definition in 3D: A bias-free, automated method
Source: PLoS One. 2023 Nov 27;18(11):e0294528. doi: 10.1371/journal.pone.0294528 (PMC10681257; doi:10.1371/journal.pone.0294528)

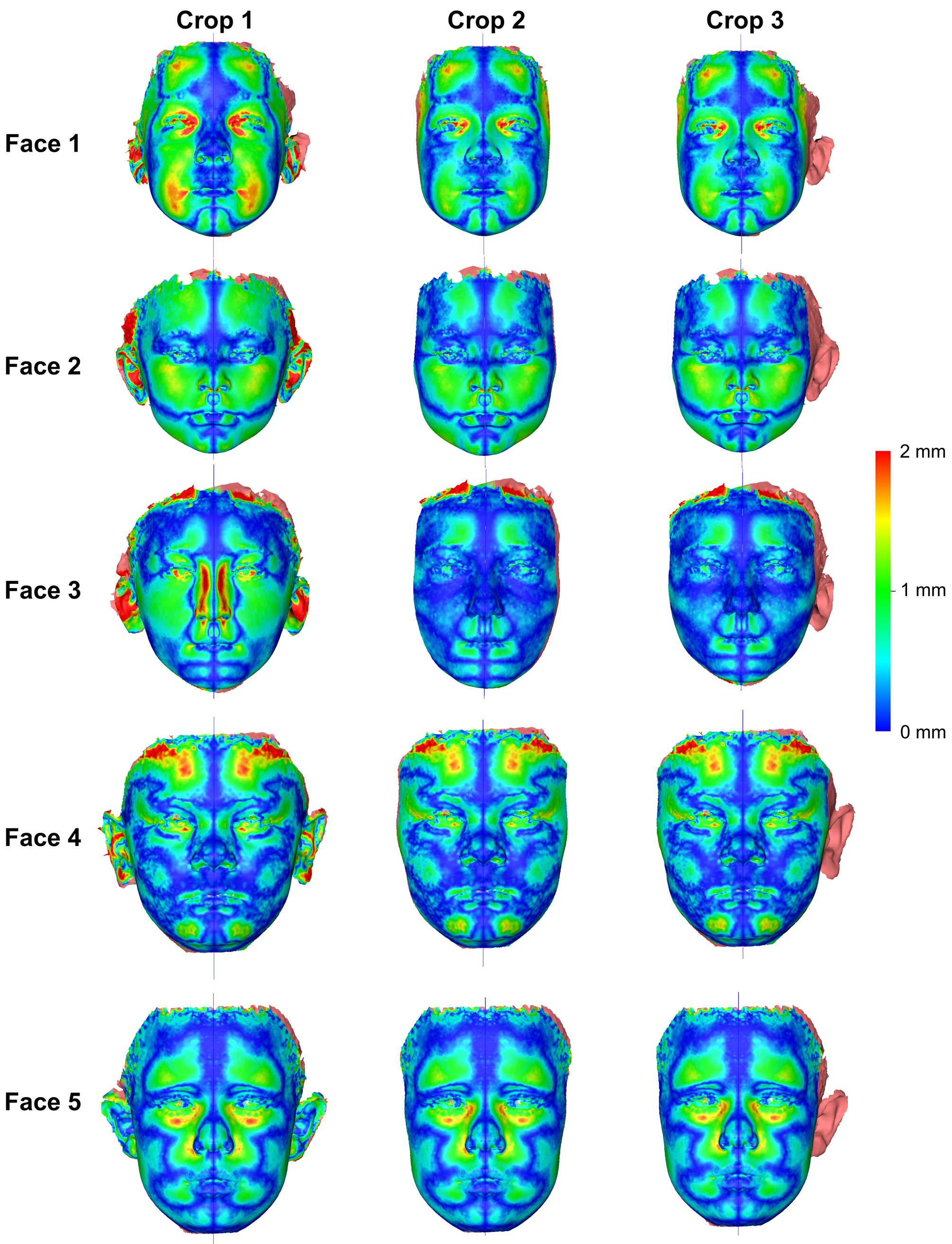

Supplement: S1 Fig — These were generated through best-fit approximation of the surface models of five individuals with their mirrored duplicates (Faces 1–5). (TIF) [file pone.0294528.s003.tif]

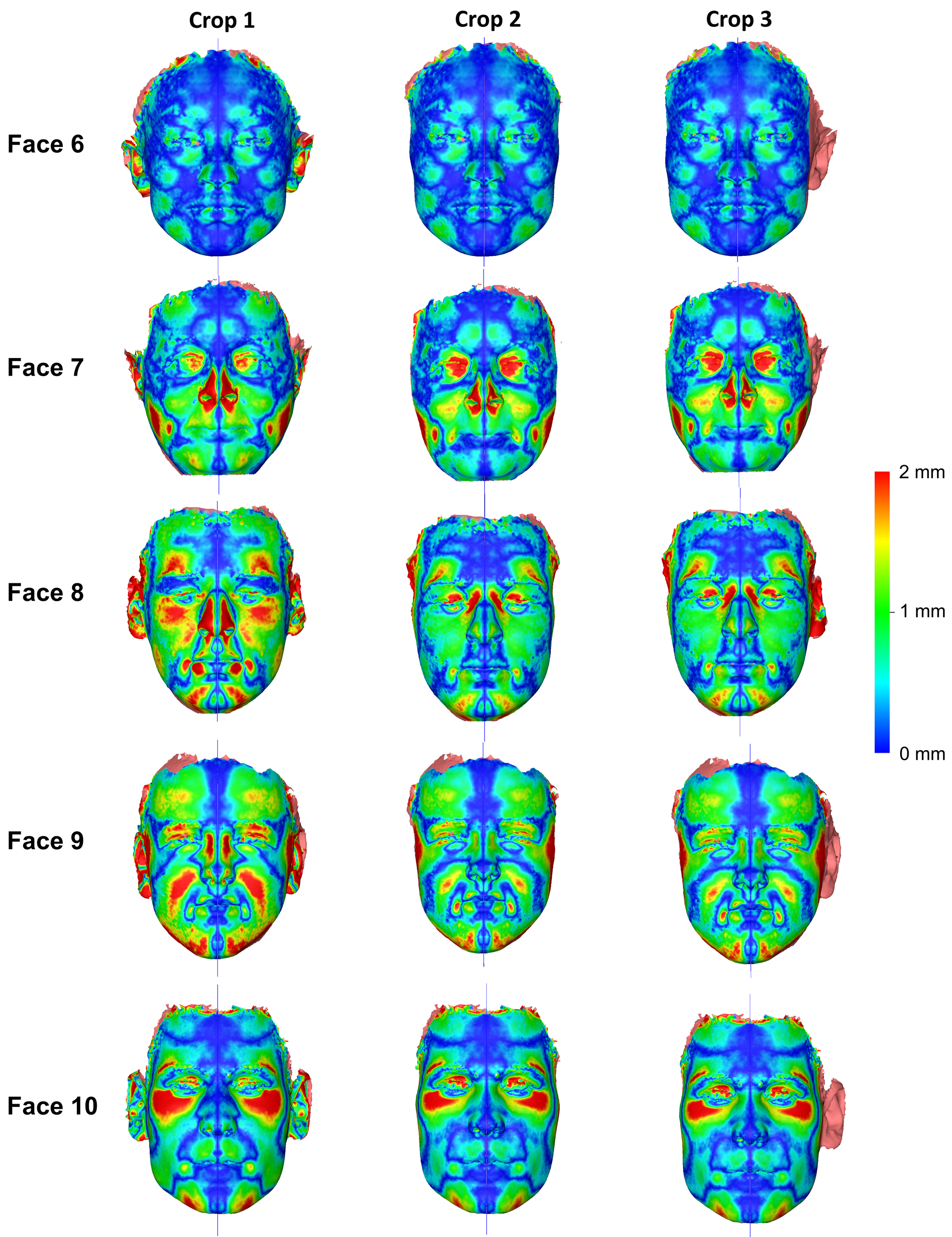

Supplement: S2 Fig — These were generated through best-fit approximation of the surface models of five individuals with their mirrored duplicates (Faces -10). (TIF) [file pone.0294528.s004.tif]

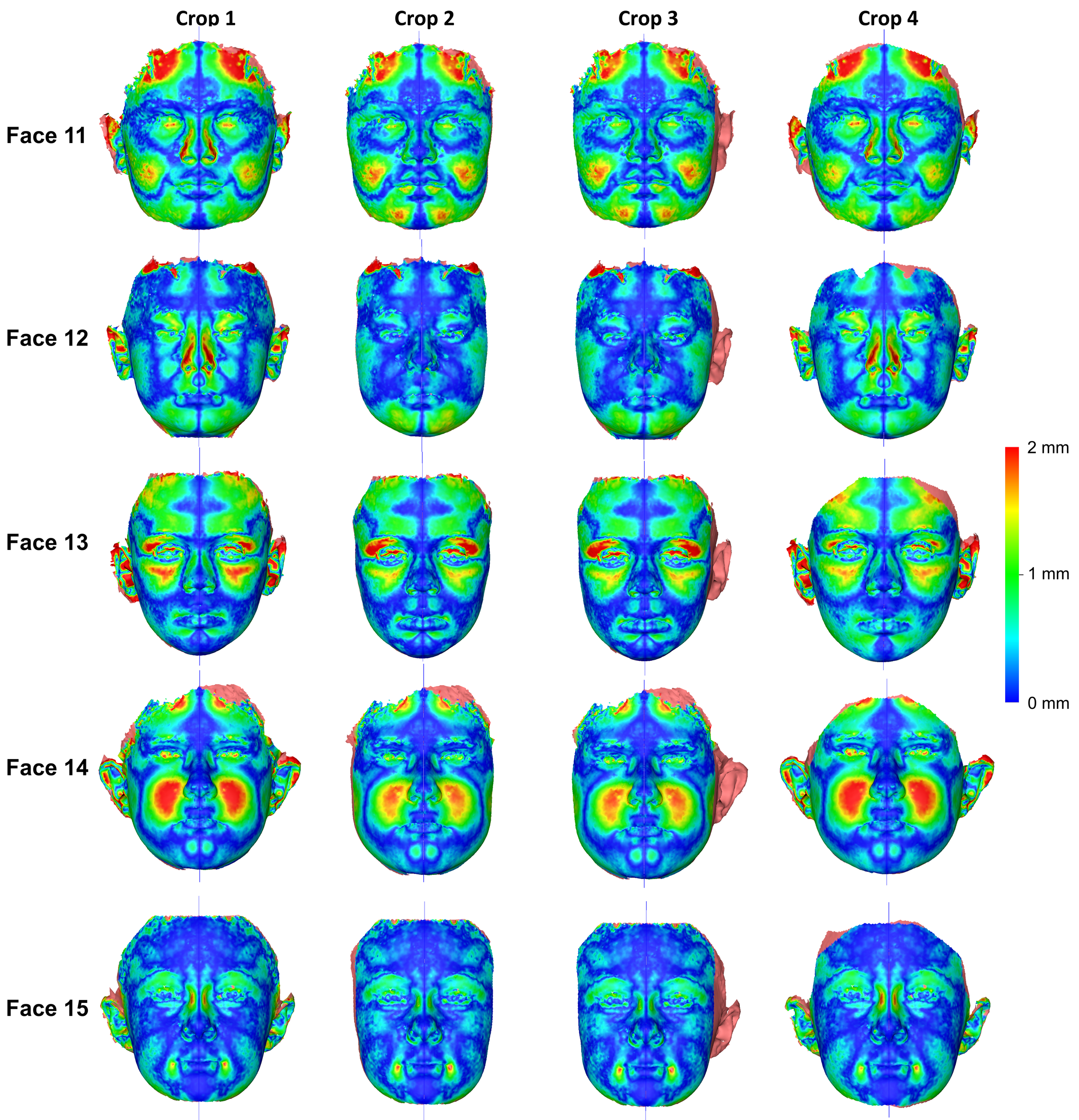

Supplement: S3 Fig — These were generated through best-fit approximation of the surface models of five individuals with their mirrored duplicates (Faces 10–15). (TIF) [file pone.0294528.s005.tif]

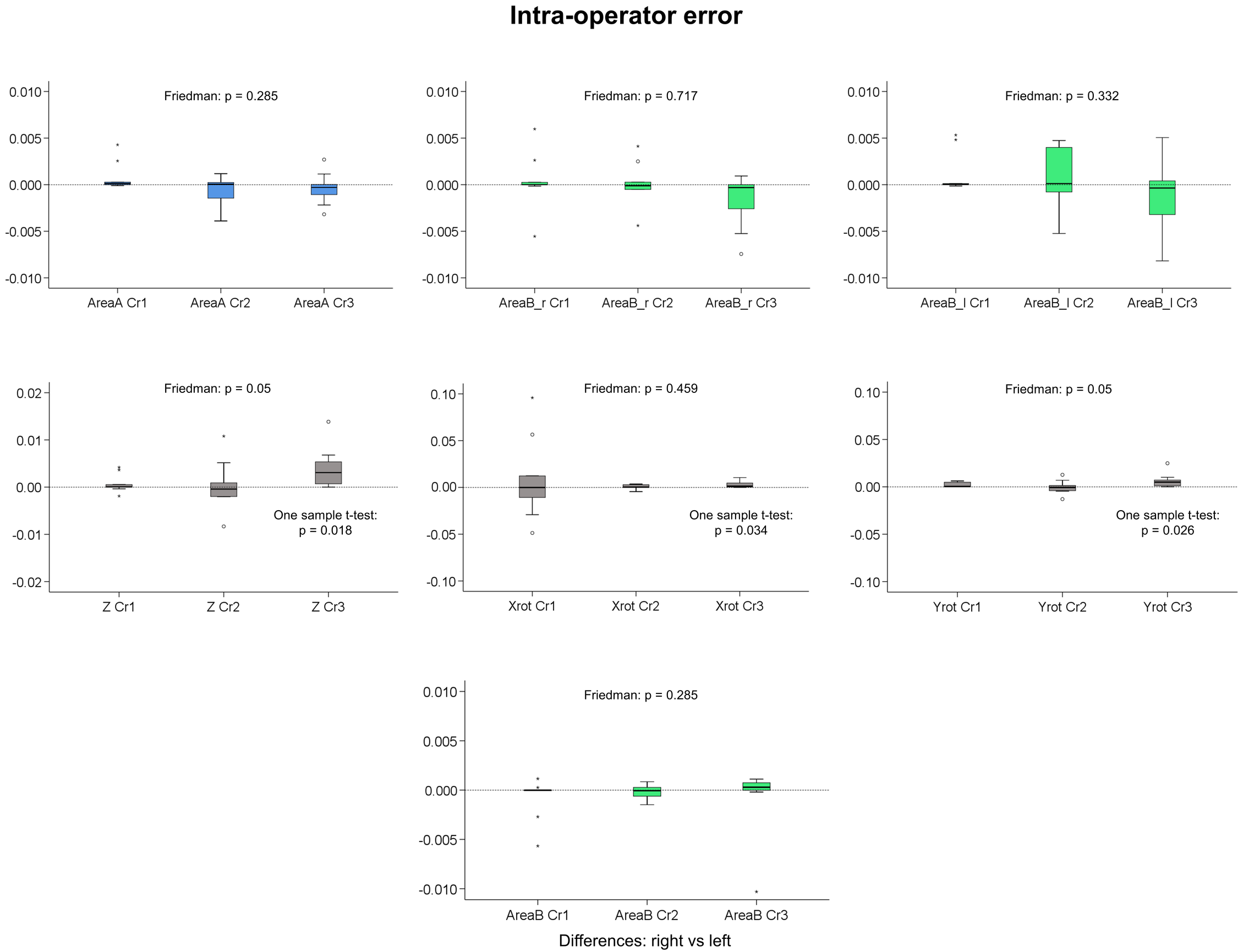

Supplement: S4 Fig — Box plots showing the intra-operator error in asymmetry assessments (upper and lowest row) and in midsagittal plane generation (middle row; Z: lateral movement in mm; Xrot: rotation around the anteroposterior axis in°; Yrot: rotation around the vertical axis in°). Outliers are shown as black circles or stars in more extreme cases. rot: rotation, Cr: Crop. (TIF) [file pone.0294528.s006.tif]

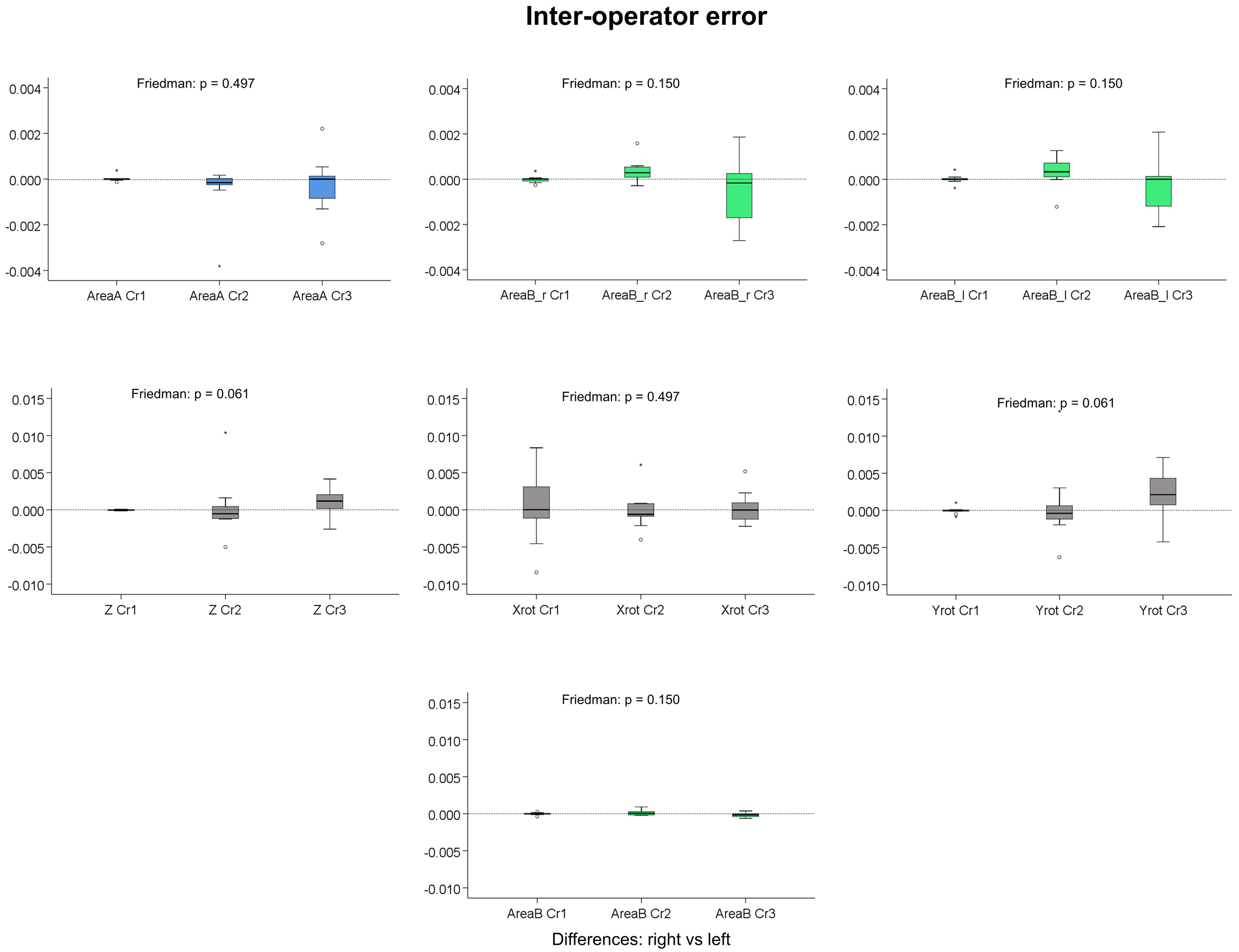

Supplement: S5 Fig — Box plots showing the inter-operator error in asymmetry assessments (upper and lowest row) and in midsagittal plane generation (middle row; Z: lateral movement in mm; Xrot: rotation around the anteroposterior axis in°; Yrot: rotation around the vertical axis in°). Outliers are shown as black circles or stars in more extreme cases. rot: rotation, Cr: Crop. (TIF) [file pone.0294528.s007.tif]

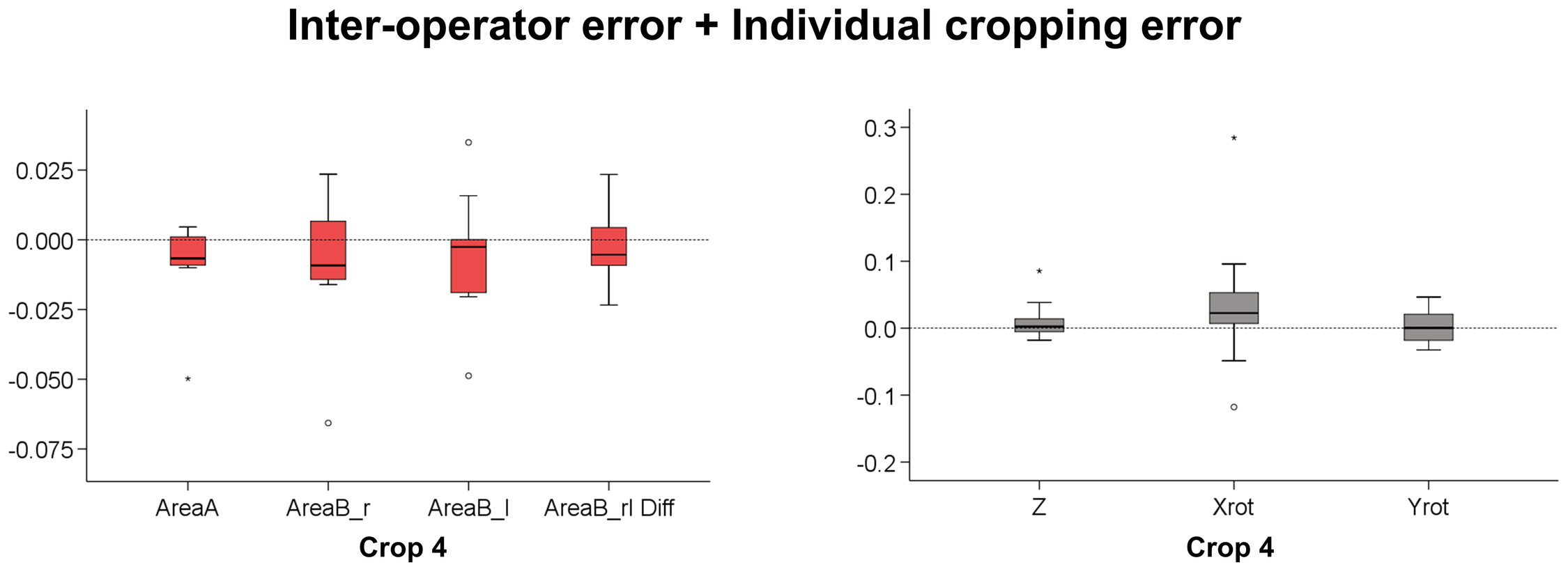

Supplement: S6 Fig — Box plots showing the inter-operator error in asymmetry assessments (left side, Friedman test: p > 0.05) and in midsagittal plane generation (right side; Z: lateral movement in mm; Xrot: rotation around the anteroposterior axis in°; Yrot: rotation around the vertical axis in°; one sample t-test: p > 0.05) with Crop 4, where each facial surface was individually cropped by each operator. Outliers are shown as black circles or stars in more extreme cases. rot: rotation, Cr: Crop. (TIF) [file pone.0294528.s008.tif]

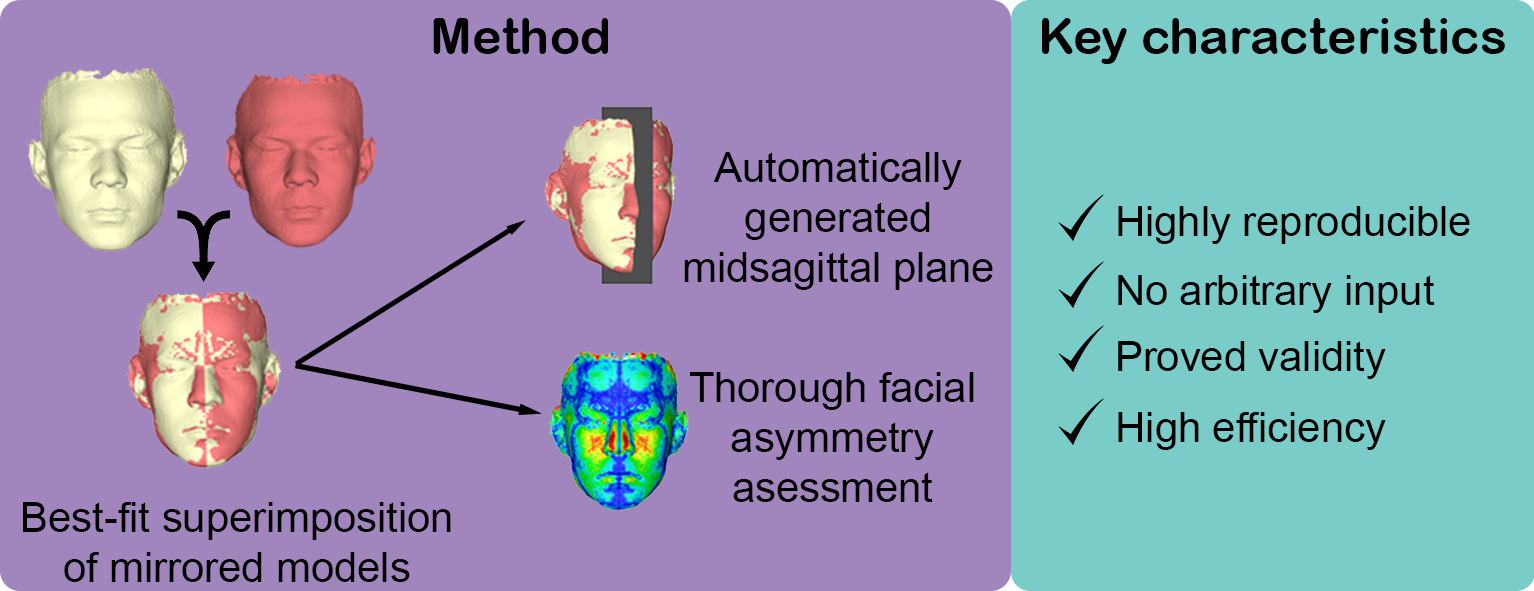

Supplement: S1 Graphical abstract — (TIF) [file pone.0294528.s009.tif]
